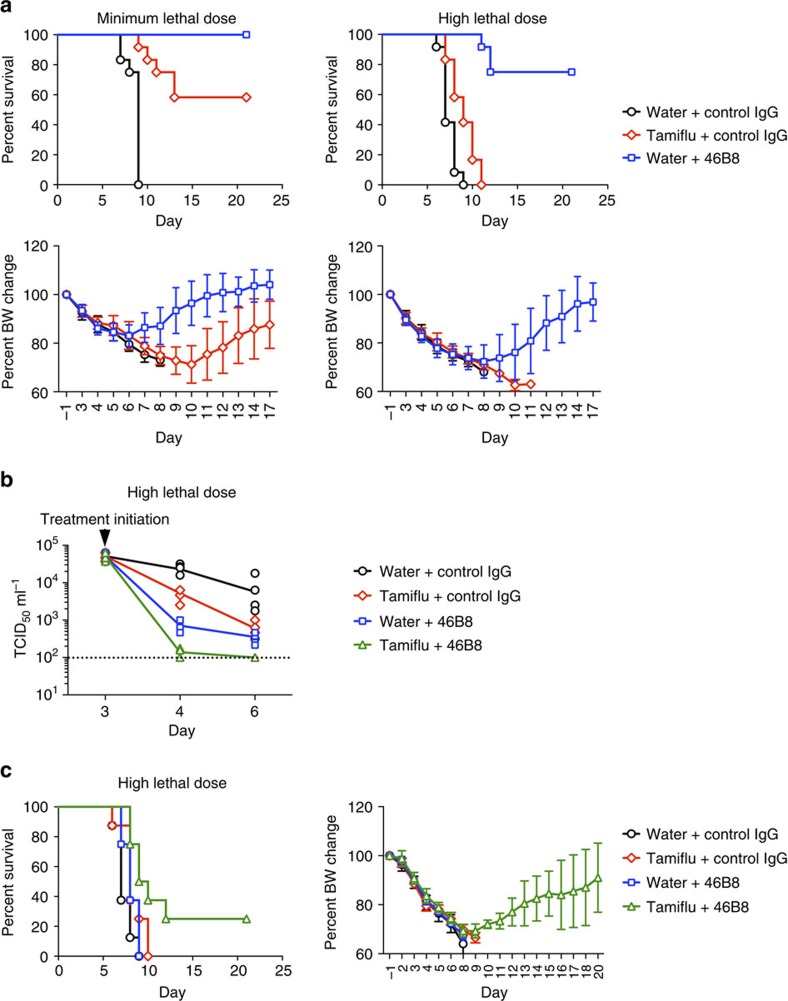# Corrigendum: A broadly protective therapeutic antibody against influenza B virus with two mechanisms of action

**DOI:** 10.1038/ncomms15779

**Published:** 2017-05-25

**Authors:** Ning Chai, Lee R. Swem, Summer Park, Gerald Nakamura, Nancy Chiang, Alberto Estevez, Rina Fong, Lynn Kamen, Elviza Kho, Mike Reichelt, Zhonghua Lin, Henry Chiu, Elizabeth Skippington, Zora Modrusan, Jeremy Stinson, Min Xu, Patrick Lupardus, Claudio Ciferri, Man-Wah Tan

Nature Communications 8: Article number: 14234; DOI: 10.1038/ncomms14234 (2017); Published: 01 19 2017; Updated: 05 25 2017

This Article contains errors in Fig. 5c. The blue squares should be labelled ‘Water+46B8' and the green triangles should be labelled ‘Tamiflu+46B8'. The correct version of Fig. 5 appears below as Fig. 1.

## Figures and Tables

**Figure 1:**