# Supplementary material for: A broadly protective therapeutic antibody against influenza B virus with two mechanisms of action
Source: Nat Commun. 2017 Jan 19;8:14234. doi: 10.1038/ncomms14234 (PMC5253702; doi:10.1038/ncomms14234)
Supplement: Supplementary Information — Supplementary Figures and Supplementary Tables [file ncomms14234-s1.pdf]

Supplementary Figure 1

a

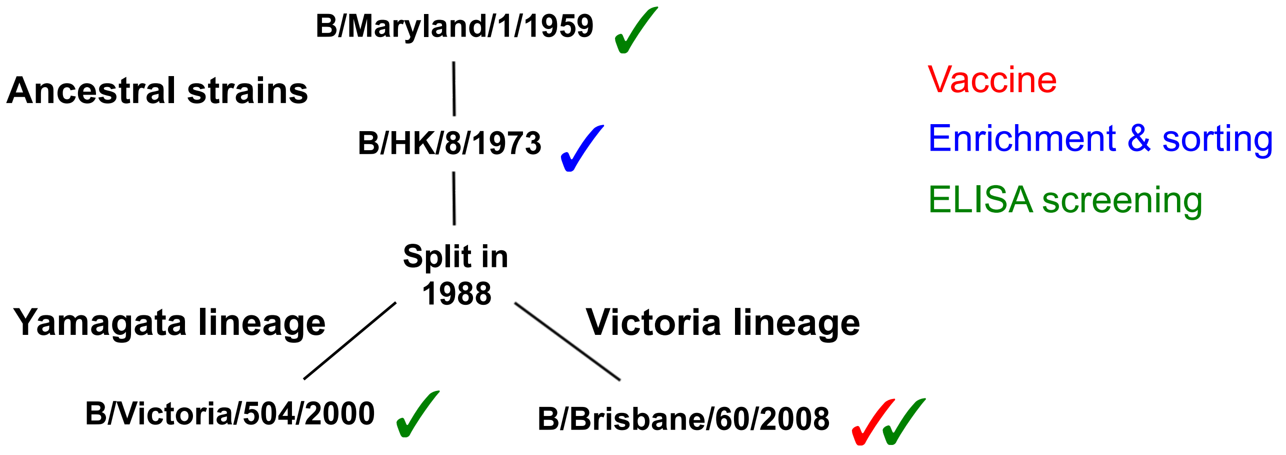

b

Number of sorted plasmablasts	2018
Number of mAbs binding all influenza B HAs tested	99
Number of mAbs neutralizing multiple influenza B viruses from both lineages and the ancestral strains	3
Number of mAbs neutralizing all influenza B viruses tested	1

Supplementary Figure 1. Antibody discovery summary. (**a**) HA proteins from the ancestral strains, the Yamagata lineage or the Victoria lineage of influenza B viruses (IBV) were used at different steps of the antibody discovery procedure. Color code: red (vaccine strain), blue (strain used for enrichment and sorting of IBV HA-specific plasmablasts), green (strains used for ELISA screening). (**b**) Number of plasmablasts or mAbs at different steps of the antibody discovery process. The only mAb that neutralized all influenza B viruses tested is 46B8.

Supplementary Figure 2

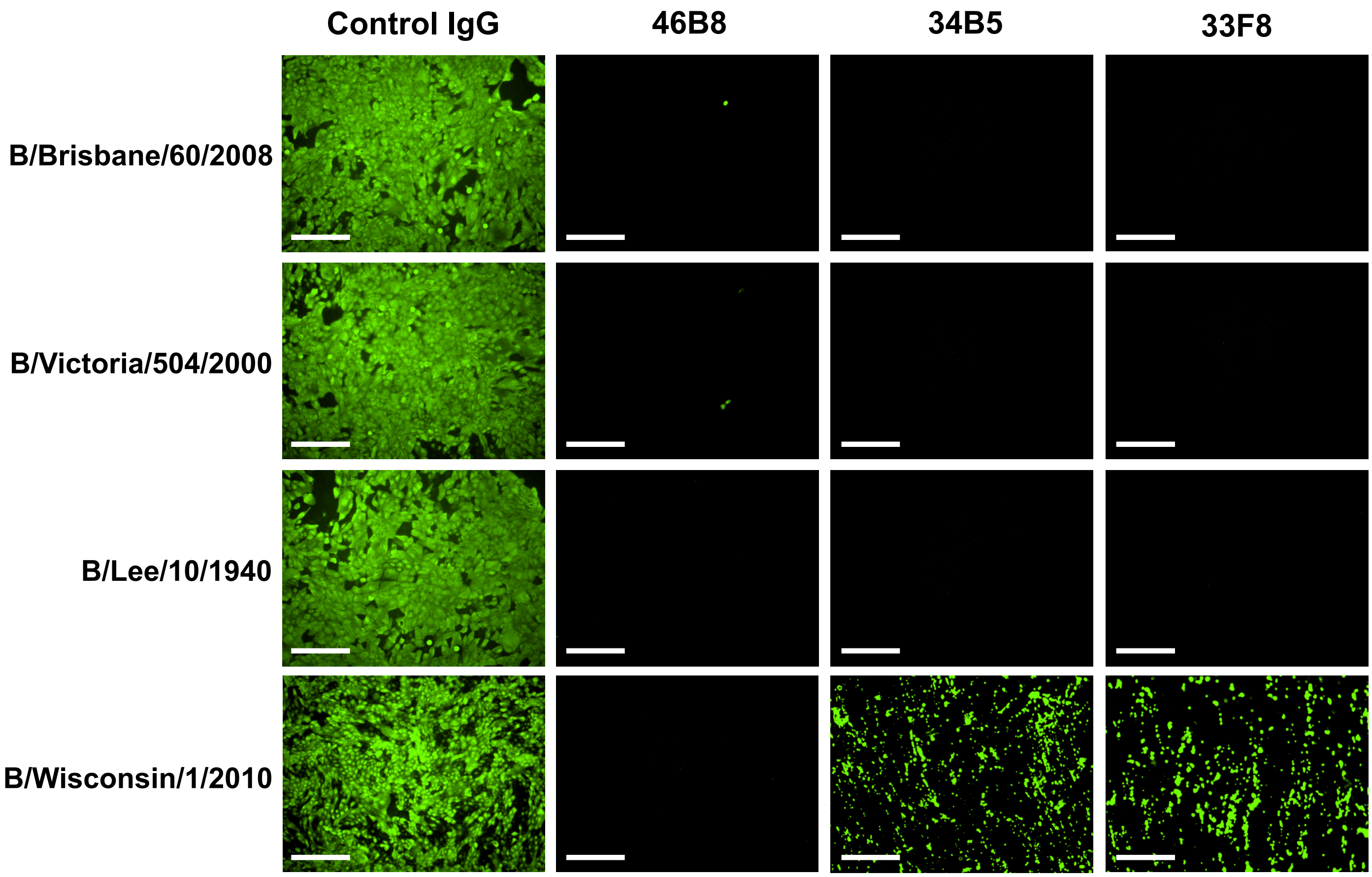

Supplementary Figure 2. Three broadly neutralizing mAbs. B/Brisbane/60/2008 (Victoria lineage), B/Victoria/504/2000 (Yamagata lineage), B/Lee/10/1940 (ancestral strain) or B/Wisconsin/1/2010 (Yamagata lineage) was incubated with 50 μg/ml of either a control IgG, 46B8, 34B5 or 33F8 prior to infection of MDCK cells. At 16 hr post-infection, cells were stained for IBV nucleoprotein (NP). Virus-infected cells are in green color. Scale bar is 200 μm.

Supplementary Figure 3

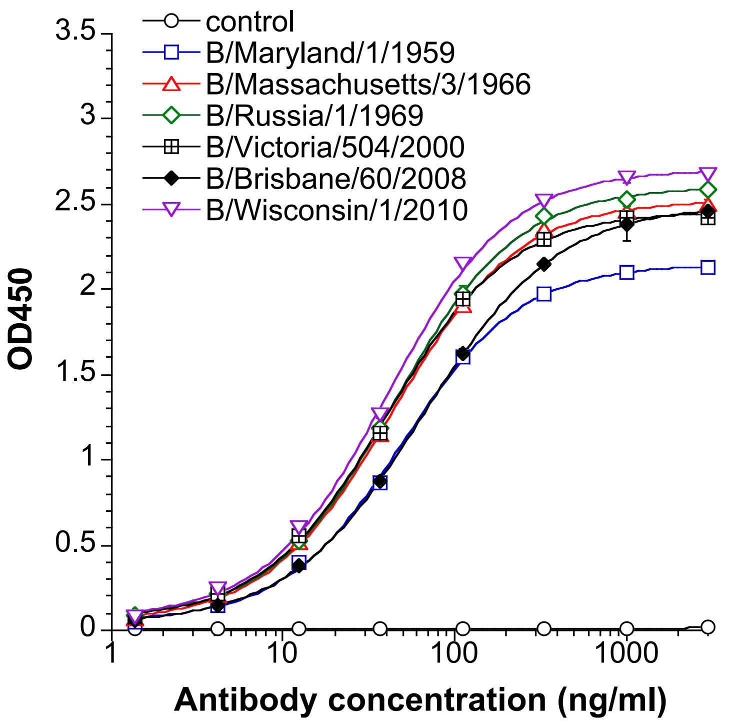

Supplementary Figure 3. 46B8 binds to all influenza B HAs tested. Lysates of 293T cells expressing influenza B HAs were coated on ELISA plates and tested for 46B8 binding. Binding as a function of absorbance at 450 nm (OD450) was plotted against the antibody concentrations. The assay was done in duplicate. Control: mock transfected cells. Maryland/1959, Massachusetts/1966 and Russia/1969 are ancestral influenza B strains; Victoria/2000 and Wisconsin/2010 are from the Yamagata lineage; Brisbane/2008 is from the Victoria lineage. (mean and s.e.m.)

Supplementary Figure 4

a

	Control IgG	34B5	46B8
B/Victoria/504/2000	> 125	0.2	> 125
B/Wisconsin/1/2010	> 125	50	> 125

b

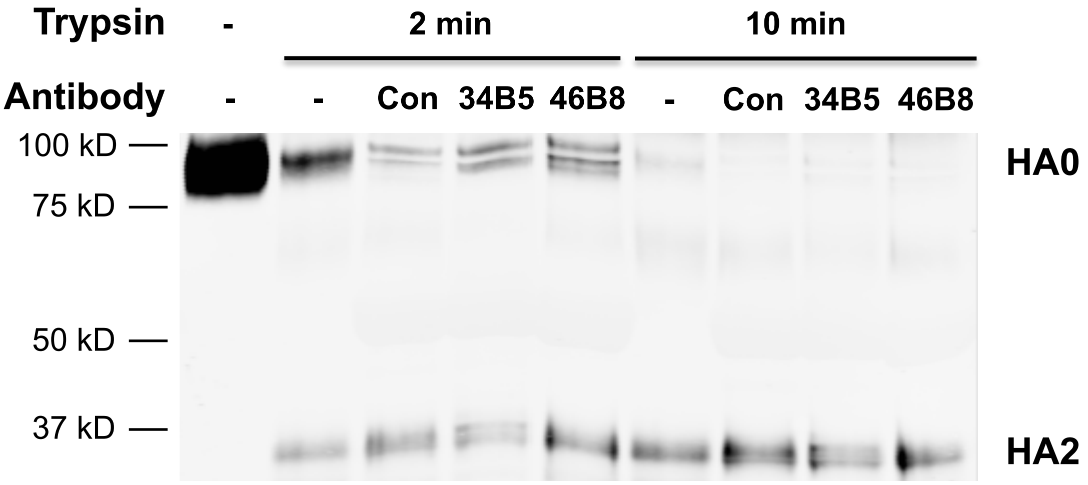

Supplementary Figure 4. 46B8 does not block viral attachment or HA0 activation. (**a**) Hemagglutination inhibition (HI) assay. The HI titers of a control IgG, 34B5 or 46B8 were determined for B/Victoria/504/2000 and B/Wisconsin/1/2010 on turkey red blood cells. Results are shown in μg/ml. (**b**) Recombinant soluble B/HK/8/1973 HA protein was incubated with a control IgG, 34B5 or 46B8 prior to trypsin digestion of 2 or 10 min. Samples were subjected to Western blot analysis with a monoclonal antibody that recognizes the HA2 subunit of influenza B viurs HA. Con: control IgG. Positions of molecular markers are shown at left.

Supplementary Figure 5

a

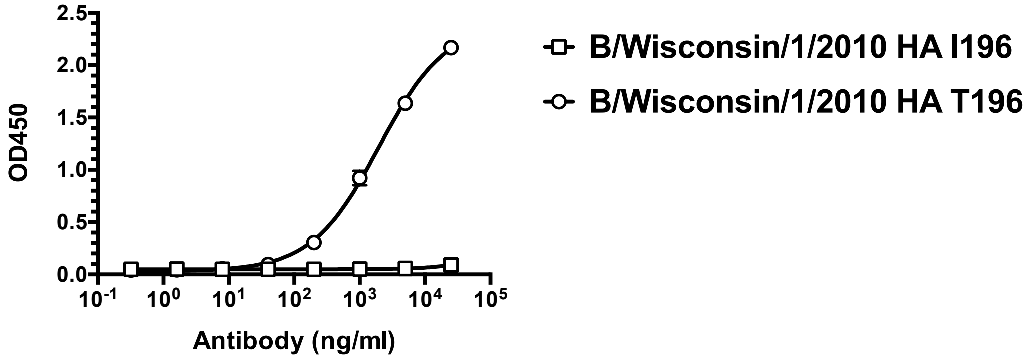

b

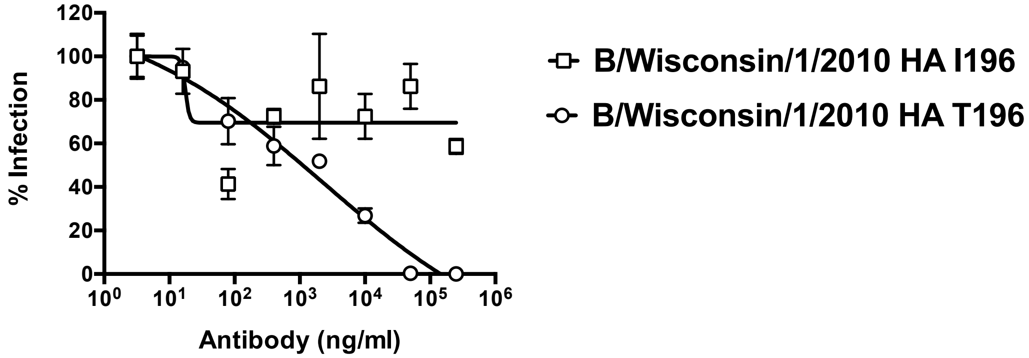

Supplementary Figure 5. 34B5 recognizes B/Wisconsin/1/2010 HA T196 but not I196. (**a**) Soluble B/Wisconsin/1/2010 HA I196 or T196 protein was coated on ELISA plates and tested for 34B5 binding. Binding as a function of absorbance at 450 nm (OD450) was plotted against the antibody concentrations. The assay was done in duplicate. (**b**) HIV pseudotype virus bearing the B/Wisconsin/1/2010 HA I196 or T196 was tested for neutralization with 34B5 on 293T cells. The numbers of infected cells were determined by counting GFP-positive cells. Infection of each virus was normalized to the number of infected cells at the lowest antibody concentration. Percent of infection was plotted against the antibody concentrations. The assay was done in duplicate. (**a,b**: mean and s.e.m.)

Supplementary Figure 6

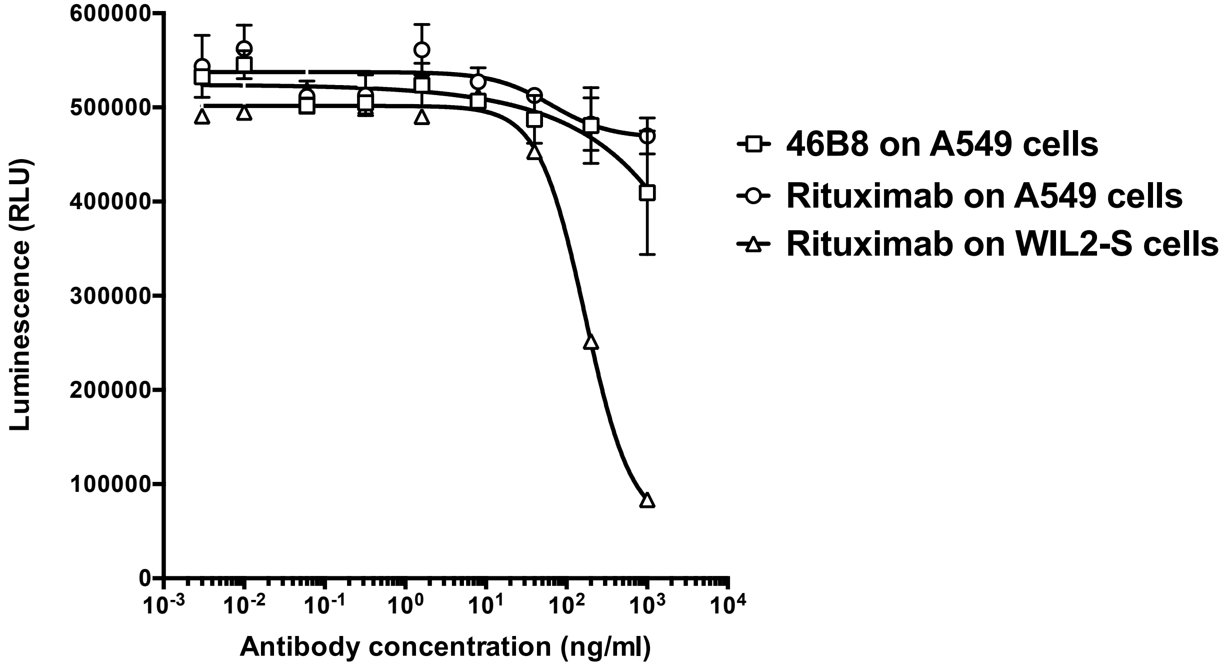

Supplementary Figure 6. 46B8 does not induce CDC in vitro. A549 cells infected with B/Brisbane/60/2008 were labeled with 46B8 or Rituximab (negative control) followed by incubation with complement to allow cell lysis. As a positive control, WIL2-S cells (expressing CD20) were labeled with Rituximab prior to complement exposure. CellTiter-Glo reagent was then added to detect ATP in the remaining live cells that were not lysed by CDC. Luminescence signals from live cells were plotted against antibody concentrations. The assay was done in duplicate. RLU, random luminescence unit. (mean and s.e.m.)

Supplementary Figure 7

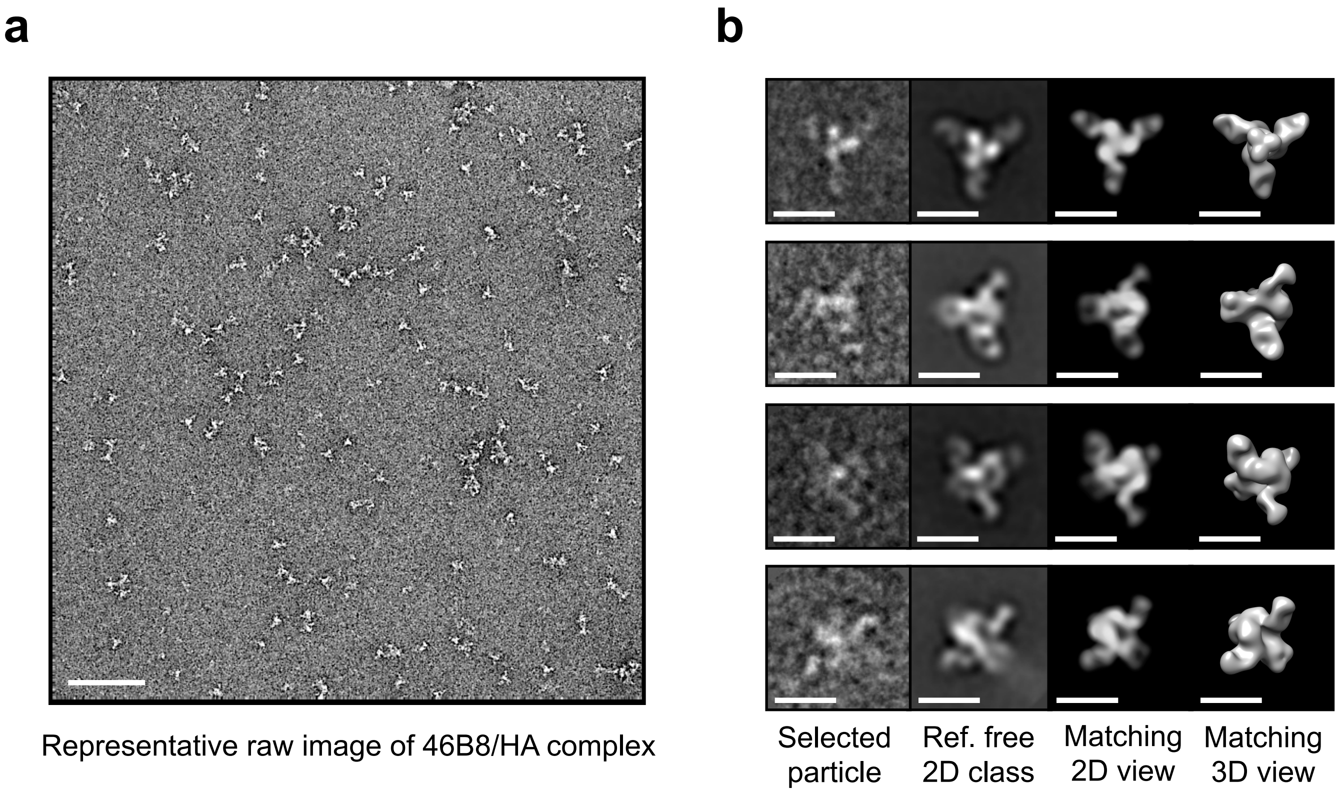

Supplementary Figure 7. Raw electron microscopy (EM) images and reconstruction of 46B8/HA complex. Complex of 46B8 Fab and B/Victoria/504/2000 HA ectodomain was purified and subjected to negative stain EM imaging and reconstruction. (**a**) Representative raw image collected on a Tecnai-12 Biotween operated at 120 kV and a magnification of 62,000x. Scale bar is 100 nm. (**b**) Comparison between a representative particle (Selected particle), the corresponding reference free 2D class (Ref. free 2D class), the 2D projection of the obtained reconstruction (Matching 2D view) and the 3D model obtained (Matching 3D view) from four independent views. Scale bar is 10 nm.

Supplementary Figure 8

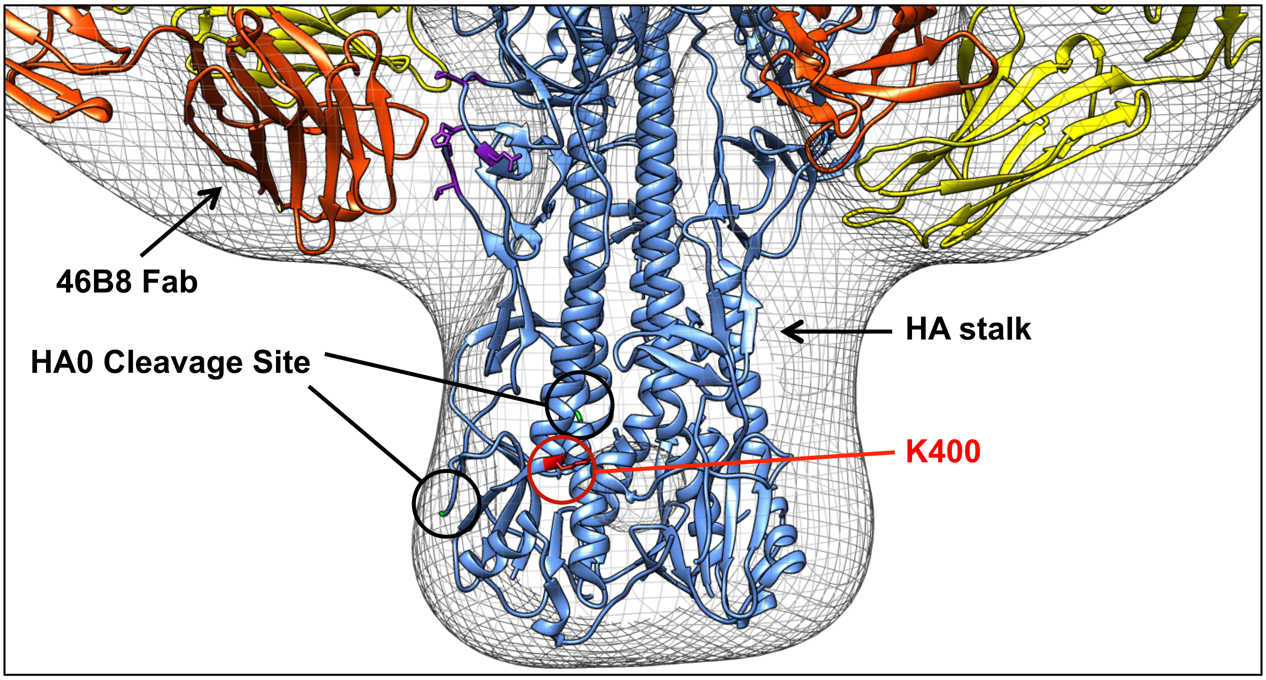

Supplementary Figure 8. Residue Lys400 (K400) and the HA0 cleavage site are distant from the 46B8-binding region on HA. Negative stain EM reconstruction of 46B8 Fabs in complex with the B/Victoria/504/2000 HA trimer, as in Fig. 3a. The last residue of HA1 and first residue of HA2 resolved in the structure (PDB ID 4M40) after HA0 cleavage are shown in green inside black circles; residue K400 is in red inside a red circle.

Supplementary Figure 9

a

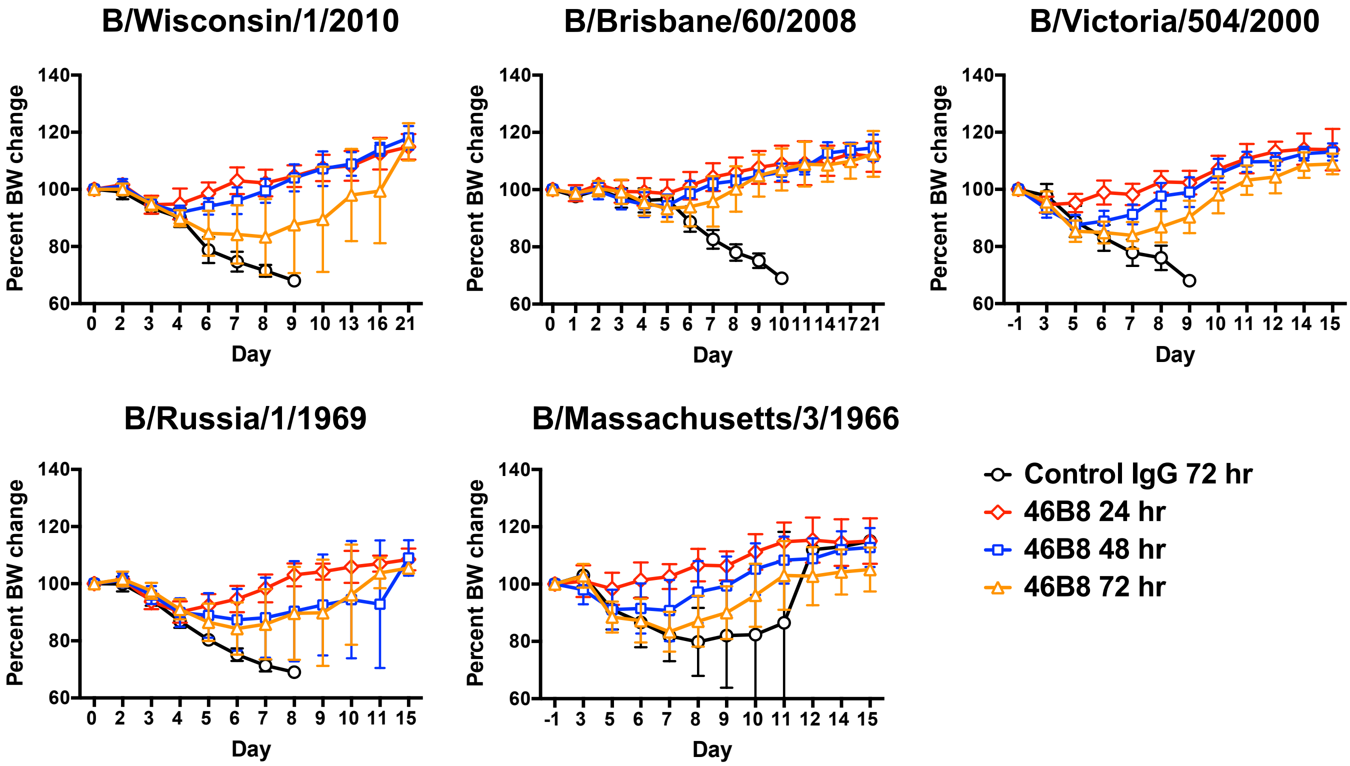

b

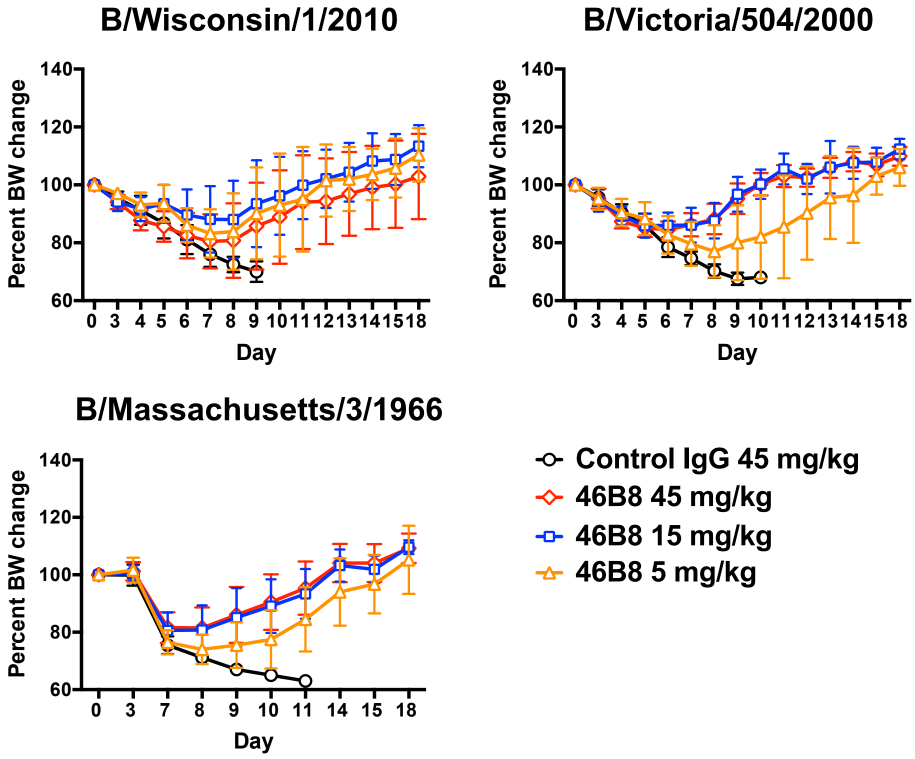

Supplementary Figure 9. Percentage of average body weight (BW) change in influenza B mouse infection models. (**a**) DBA/2J mice were infected intranasally with a minimum lethal dose of B/Wisconsin/1/2010, B/Brisbane/60/2008, B/Victoria/504/2000, B/Russia/1/1969 or B/Massachusetts/3/1966. At 24, 48 or 72 hr post-infection, mice received a single treatment of 46B8 or a control IgG intravenously at 15 mg/kg. Percent of average BW of survived mice as compared to the average pre-infection weight was plotted. Each group contains 8 mice. (**b**) DBA/2J mice were infected intranasally with a minimum lethal dose of B/Wisconsin/1/2010, B/Victoria/504/2000 or B/Massachusetts/3/1966. At 72 hr post-infection, mice received a single treatment of 46B8 or a control IgG intravenously at 5, 15 or 45 mg/kg. Percent of average BW of survived mice as compared to the average pre-infection weight was plotted. Each group contains 8 mice. (**a,b**: mean and s.d.)

Supplementary Figure 10

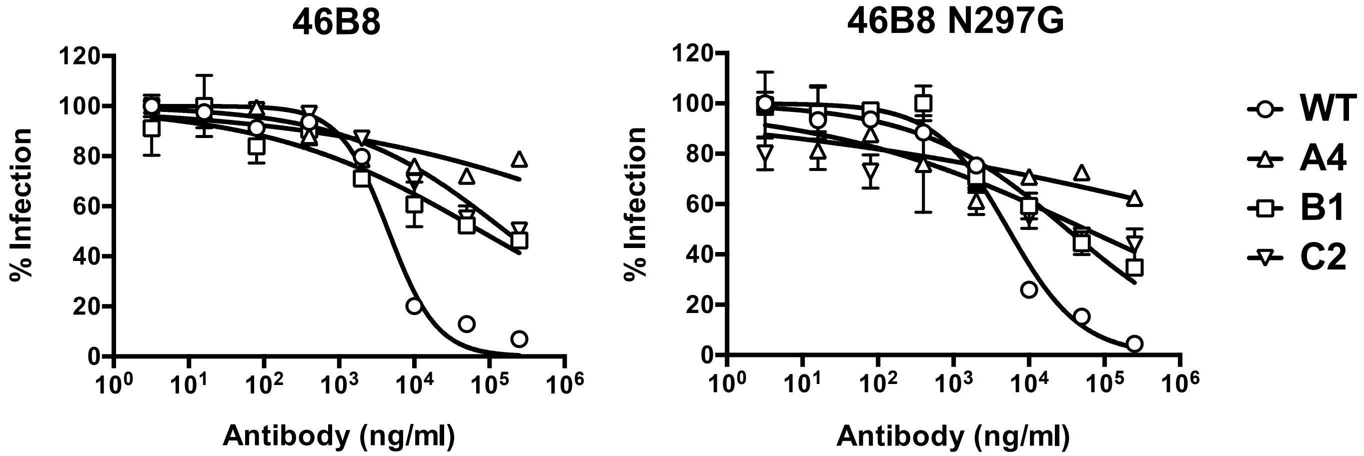

Supplementary Figure 10. Partial resistance of B1 and C2 mutant viruses to 46B8 and 46B8 N297G. WT or mutant B/Brisbane/60/2008 viruses were incubated with serial dilutions of 46B8 or 46B8 N297G ranging from 0.0032 to 250 μg/ml. Cells were incubated with the virus-antibody mixture for 16 hours prior to immuno-staining with anti-IBV NP and Hoechst 33342. The percentages of infected cells for each virus were normalized to the value at the lowest antibody concentration. The assay was done in triplicate. (mean and s.e.m.)

Supplementary Figure 11

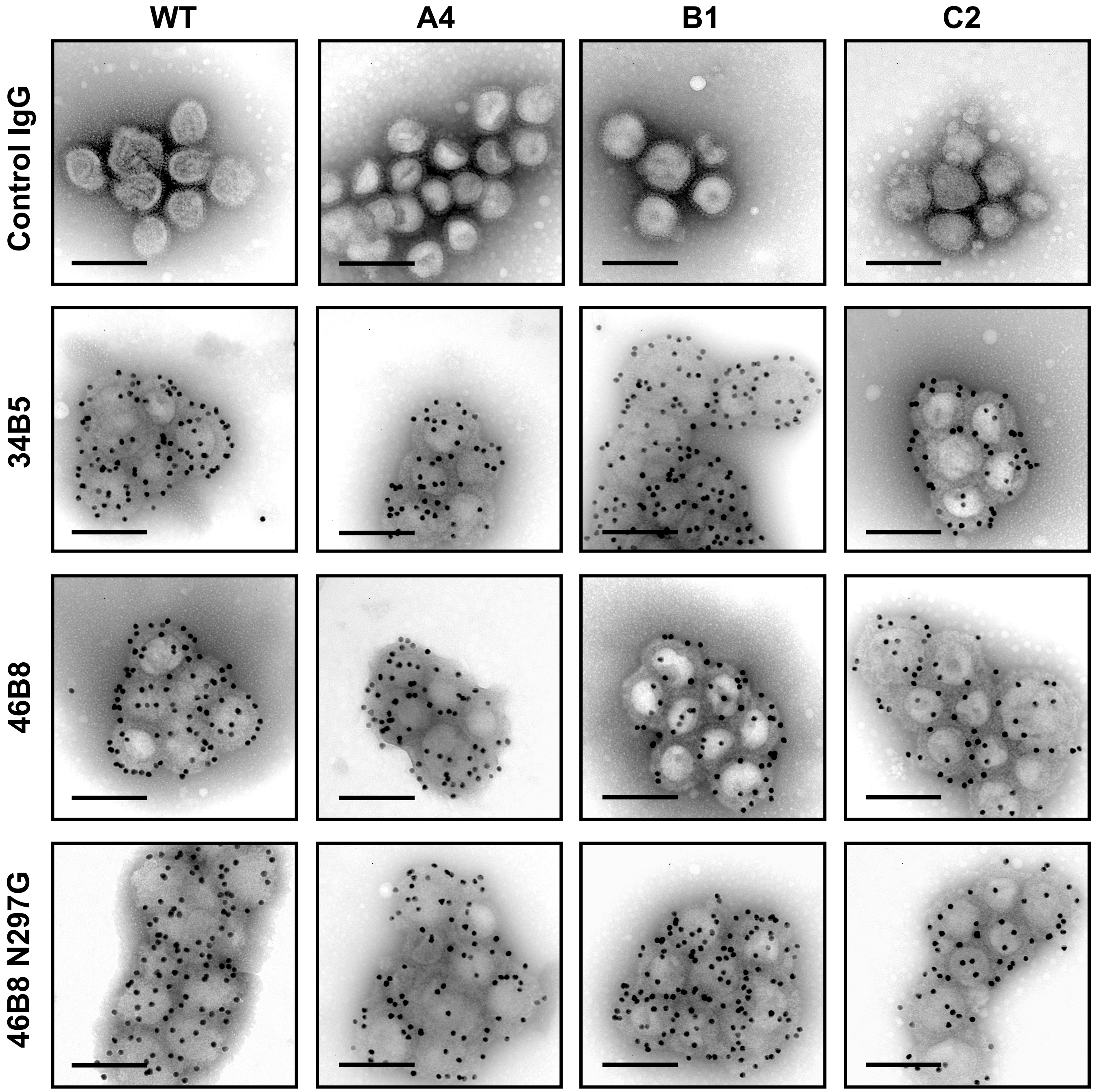

Supplementary Figure 11. Binding of mAbs to WT or mutant B/Brisbane/60/2008 viruses. Immunogold EM images of the WT or mutant B/Brisbane/60/2008 viruses bound by a control IgG, 34B5, 46B8 or 46B8 N297G. Scale bar is 200 nm.

Supplementary Figure 12

a

b

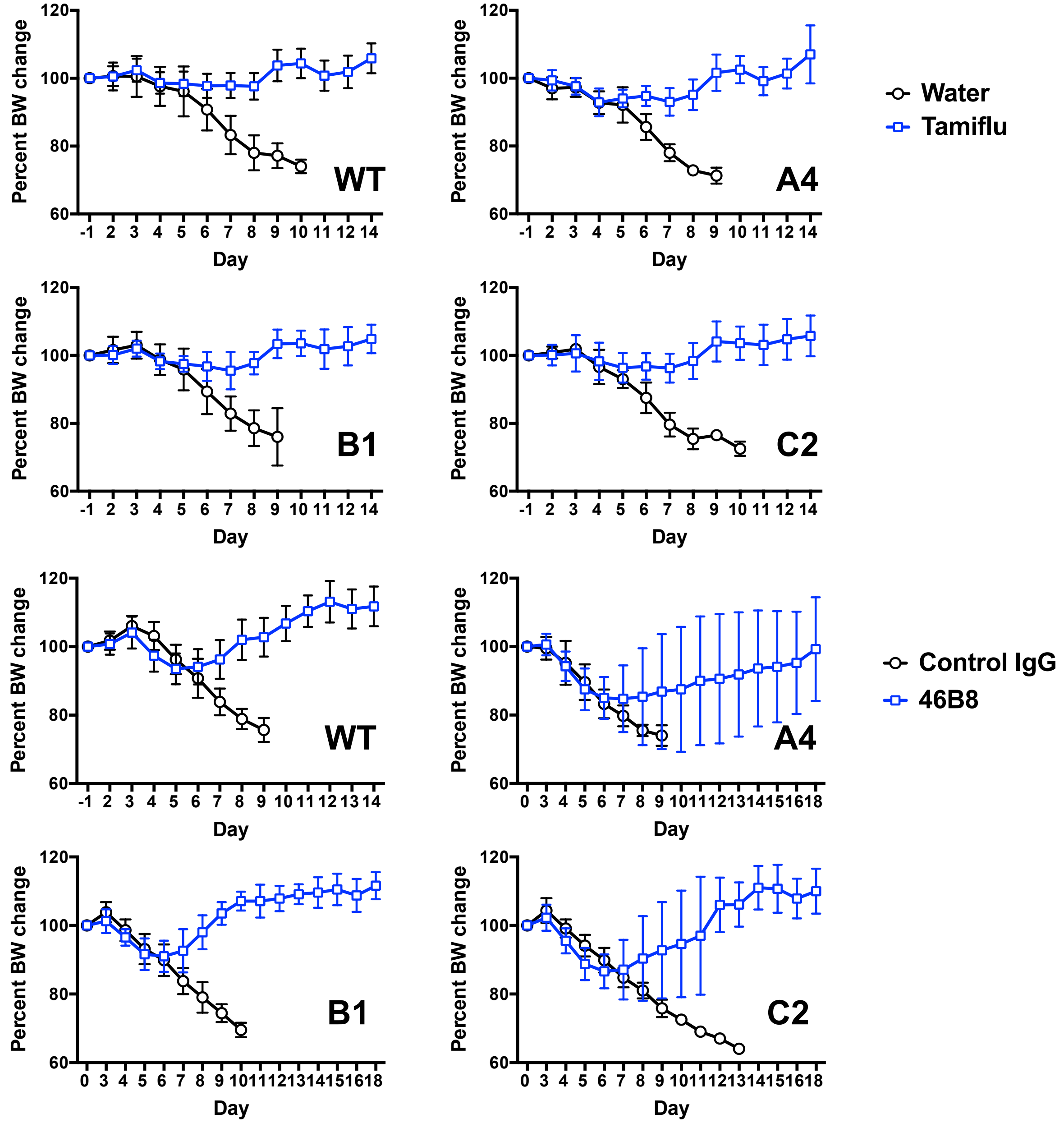

Supplementary Figure 12. Percentage of average BW change in mice infected with WT or mutant B/Brisbane/60/2008 viruses. DBA/2J mice were infected intranasally with a minimum lethal dose of the WT or mutant B/Brisbane/60/2008 viruses. (**a**) At 48 hr post-infection, mice received Tamiflu orally at 100 mg/kg twice a day for 5 days. (**b**) At 72 hr post-infection, mice received a single treatment of 46B8 or a control IgG intravenously at 15 mg/kg. Percent of average BW of survived mice as compared to the average pre-infection weight was plotted. Each group contains 8 mice. (**a**,**b**: mean and s.e.m.)

Supplementary Table 1

Mutations in non-HA viral proteins of B/Brisbane/60/2008
WT and resistant viruses

Gene	AA Pos	Ref AA	WT	A4	B1	C2
**PB1**	719	H	R:66.7%<br>H:33.3%	-	-	-
**PB2**	65	D	N:54.3%<br>D:45.3%	-	-	-
**PB2**	185	R	-	K:99.7%	-	-
**PB2**	303	I	-	-	M:100.0%	M:99.9%
**PB2**	582	E	-	-	G:99.9%	G:100.0%
**PA**	321	D	N:67.1%<br>D:32.9%	-	-	-
**PA**	338	K	-	-	-	R:99.9%
**PA**	625	V	-	A:98.7%<br>V:1.2%	A:100.0%	-
**NA**	215	H	-	-	L:59.6%<br>H:40.0%	-
**BM2**	86	I	-	-	M:55.6%<br>I:44.4%	-
**NS1**	117	Y	-	-	-	H:74.5%<br>Y:25.3%

Amino acid substitutions detected at the dominant level (> 50%) in the viruses with respect to the reference sequences. All numberings start from the N-terminal methionine. A dash indicates that no variants were detected at > 50% at the corresponding position relative to the Ref AA. Abbreviations: AA Pos, amino acid position; Ref AA, reference amino acid from GenBank.
